# Genistein as a dietary supplement; formulation, analysis and pharmacokinetics study

**DOI:** 10.1371/journal.pone.0250599

**Published:** 2021-04-27

**Authors:** Alexandra Mamagkaki, Ioannis Bouris, Panagiotis Parsonidis, Ioanna Vlachou, Maria Gougousi, Ioannis Papasotiriou

**Affiliations:** 1 Research Genetic Cancer Centre S.A., Florina, Greece; 2 Research Genetic Cancer Centre International GmbH Headquarters, Zug, Switzerland; Cairo University, EGYPT

## Abstract

The objective of this study is to improve and optimize the formulation of Genistein in capsules in order to result in a better pharmacokinetic profile comparing to existing commercial products. In order to do this, five different formulations of Genistein capsules were developed and examined by reviewing their disintegration and dissolution properties. Furthermore, flowability of the powder along with potent incompatibilities between Genistein and its excipients were monitored through their thermal properties. The final formulation of Genistein was quantified using HPLC analysis and then its stability was evaluated thoroughly in real time and accelerated conditions. Finally, with the target to have a product with actual results, *in vitro* and *in vivo* studies were conducted. The final product proved to have better results in disintegration and dissolution. Moreover, R.G.C.C.’s capsules exhibited enhanced action in human cell lines as well as impressive pharmacokinetic results in animal models. The *in vitro* results showed an advantage of the R.G.C.C. product compared to the commercial one, whereas its maximum concertation *in vivo* was determined 34% higher than the commercial one.

## Introduction

The regular use of dietary supplements is a worldwide phenomenon. This comes along with the evidence supporting that daily nutrition, mental balance, healthy habits and general wellness are few of the aspects that influence our lifestyle. By interfering to these, the prevention of serious diseases is more effective, targeted and with long term effects. The last decades many studies put in the spotlight the analysis and use of natural products, which have shown therapeutic effects, based mainly on traditional medicine. However, since dietary supplements belong to a multi-dollar industry and at the same time, guidelines and legislation are lenient, there are often brought to the market unauthorized products without adequate quality control but enthusiast claims.

Soy and soy-derived products are examples of these products, that gain everyday more and more recognition. Based on scientific data, the Food and Drug Administration recently approved a health claim for soy protein, which states that 25 g of soy protein per day as a part of a diet low in saturated fat and cholesterol may reduce the risk of heart disease. The most popular and effective group of compounds in soy and for which an important amount of studies and analysis has been conducted, is isoflavones. These polyphenols, often-called estrogenic isoflavones, act as estrogen antagonists. Studies claim that they bind to the estrogen receptor and can affect the generation of estrogen-induced responses. Apart from this, they are used for the prevention of bone diseases like osteoporosis or diseases connected to heart [1–3]. The predominant isoflavone, Genistein has been characterized for its properties thoroughly. It can induce cell differentiation, altering apoptosis, the cell cycle and angiogenesis. In addition to these, Genistein can inhibit metastasis and has shown synergy in combination with chemotherapeutic agents. Because of its activity, it is mentioned as a phytoestrogen [4–8].

Asian populations have shown interesting clinical and epidemiological data and this was the motivation for the analysis of its nutritional effect. The daily consumption of isoflavones in Asian women along with the low numbers of osteoporosis, cardiovascular disease even breast and urine cancer that they have shown, has brought the scientific community to associate isoflavone’s action against these. The most popular and important isoflavone, Genistein, has an encouraging role in hormone-related cancers. This is justified by its structure, which is analogous to 17β-estradiol and it has 4% binding affinity for ER-α and 87% for ER-β [7,9–11].

In principle, this has a practical impact on post-menopausal women since the consumption of soy can help with menopausal symptoms. Especially women, who want to avoid hormone replacement therapy and eliminate the risks associated with this, opt for this option [12].

Various studies have underlined the hormonal activity of these isoflavones. Breast Cancer [13–15], Prostate Cancer [16–21], Uterine Cancer [22], even climacteric syndrome are examples of cases where genistein, primarily, and other Isoflavones have demonstrated positive results in *in vivo* or *in vitro* studies [6,11,23–27].

Oral administration seems to be the most patient-friendly and widely compliance administration. However, it is a challenge for pharmaceutical scientists. Its low bioavailability results and the possible degradation that could happen, make its development difficult [2,28]. Capsules and tablets are the most common pharmaceutical dosage forms in this section and in the case of Isoflavones someone can find different brands and varieties in the market. The main problem with Isoflavones like Genistein, Daidzein, Glycitein etc. is that they have poor water solubility along with high membrane permeability (Class II drugs in BCS) [1,29–31].

In this study, different formulations for hard-shell capsules via dry granulation method were prepared in order to improve genistein’s absorption and pharmacokinetics. The product was verified through analytical chemistry methodologies and various quality control methods. Furthermore, *in vitro* studies of R.G.C.C’s product were conducted versus the most common commercial one and the experiments were finalized by sending R.G.C.C.’s capsules along with the commercial ones for pharmacokinetic assays on animals.

## Materials and methods

Genistein (*Sophora japonica L*.) 98% was purchased from Shaanxi Yi An Biological Technology Co.,Ltd (Xian, Shaanxi, China), and Genistein analytical standard ≥97% (standard for HPLC) was purchased from Sigma-Aldrich (Louis, MO, USA). A mixture of corn starch and pregelatinized starch was bought from Colorcon (Lower Salford Township, Pennsylvania, USA), Nu-FLOW^®^, a natural replacement for silica dioxide from Ribus Inc (St. Louis, MO 63122, USA) and Maize starch from Cargill (Schiphol, The Netherlands). Sodium dodecyl sulfate (SDS), acetonitrile, and water for HPLC were also purchased from Sigma-Aldrich (Taufkirchen, Germany). Glacial acetic and Silicon Dioxide acid were purchased from Merck KGaA (Darmstadt, Germany). The capsules that were used in this experiment were Hypromellose Capsules by Capsugel (Basel, Switzerland).

## Formulation of capsules

In order to evaluate the right type of formulation different types of recipes and manufacturing methods were examined. Each formulation was evaluated through its dissolution and disintegration properties and overall profile. At Table 1, there is an overview of the different recipes, which were all prepared with dry granulation method. The excipients used to formulate the capsules were, Starcap 1500^®^ (a mixture of corn starch and pregelatinized starch), Corn Starch, Nu-FLOW^®^ and Silica Dioxide. Two different Genistein formulation strengths were prepared in order to achieve wider market approval. The final dosage unit was decided to be hard shell capsules. Capsules were filled manually using a manual capsule filling machine of 100 capsules. Capsules of 30 mg/ cap Genistein, had an average weigh of 100 mg and net weight 60 mg. They were packed in 120 capsules per package HDPE bottles. Capsules of 125mg/ cap Genistein, had an average weight of 310 mg and new weight 250 mg. They were packed in 60 capsules per package HDPE bottles.

**Table 1:** Presentation of the different formulation recipes that were examined during this study.

Formulation Code	Genistein (%)	Excipient 1 (%)	Excipient 2 (%)	Excipient 3 (%)	Excipient 4 (%)
Ingredient					
1	50	39.5	10	0.5	-
2	50	42	7.5	0.5	-
3	50	49.5	-	o.5	-
4	50	49	-	1.0	-
5	50	39.5	10	-	0.5

### Disintegration—Dissolution

The first method for the evaluation of the drug pharmaceutical profile is disintegration. The disintegration apparatus is LB-2D by LeadTop (Ruian City, Zhejing Province China). The temperature was stable at 37°C and the disintegration time was recorded continuously during the procedure. The buffer solution that was used in this method was Phosphate Buffer Solution, pH 6.8.

Afterwards, the different formulations were evaluated based on their behavior during dissolution. Dissolution apparatus that was used here is a Basket Apparatus (USP), Model RCZ-8 by LeadTop (Ruian City, Zhejing Province, China). Each vessel contained one capsule, in a 900 mL Solution, at 37°C for 90 minutes. As reference solution each time, one vessel contained a mixture of the excipients used in each formulation at the same portions. The 5 different formulations of Genistein capsules were tested for their dissolution profile in 3% SDS aqueous solution. Aiming to fully characterize and examine the behavior of the final formulation different solutions were used, but 3% SDS aqueous solution presented the best dissolution profile. Sampling and evaluation was done as described in European Pharmacopoeia Chapters (Eu.Ph. 2.9.3).

The evaluation of the dissolution percentage is completed though UV/VIS Spectroscopy (Libra S-12 UV/VIS Spectrometer, Biochrom Cambridge UK). After each time point, samples were collected from the vessels, through the sampling port of the apparatus which is connected with syringes. The solution was filtered through 0.45 um filters (LLG, PTFE, Ø13 mm, 0,45 mm, Metrolab S.A., Athens, Greece) and placed into tubes for analysis. The analysis was done by preparing standard solutions from the samples diluted in the same solvent, targeting specific concentrations and based on a standard curve, the amount of Genistein in each tube was estimated.

### Flowability

Pharmaceutical industry utilizes powders in many different applications. Thus, a variety of methods has been developed with the aim to characterize the flow properties of these powders. The US Pharmacopoeia <1174> Powder Flow, states “it is clear that no single and simple test method can adequately characterize the flow properties of pharmaceutical powders”. One of the most common methods often reported in studies, is angle of response. Based on pharmacopoeia US <1174>, Angle of response is a characteristic related to interparticulate friction or resistance to movement between particles. However, there are a few limitations with the use of this method, even though it is widely applied in pharmaceutical industry since it can offer a valuable prediction of possible future problems during manufacturing.

There are a range of different tests methods in literature, but the most common and important experimental variables are the two following: the height of the “funnel”, through which the powder passes and the base upon the pile forms. The height of the funnel can be fixed relative to the base or can be varied as the pile forms. In addition to these, the same applies for the base of the pile, which can either be a fixed diameter or the diameter of the powder cone can vary as the pile forms. In this case, in all of the experiments it was chosen to keep the height of the funnel as a standard value and the base of the pile was measured as the pile formed. The same chapter of USP mentions the mathematical formulas the general experimental procedure with more details. In Table 2, the Flow properties and Corresponding Angles of Repose are summarized based on the USP chapter.

**Table 2:** (<USP 1174>) flow properties and corresponding angles of repose.

Flow Property	Angle of Repose (degrees)
Excellent	25–30
Good	31–35
Fair- aid not needed	36–40
Passable- may hang up	41–45
Poor- must agitate, vibrate	46–55
Very poor	56–65
Very, very poor	>66

### Differential scanning calorimetry

During pre-formulation studies, one crucial step is to examine excipients and different combinations of excipients and API for potent incompatibilities. DSC 3 (STARe System) was used from Mettler, Toledo. The software used to evaluate the results is STARe Software. Appropriate amount of samples were sealed in pierced aluminum pans 40 uL and heated from 0°C to 400°C with a scanning rate of 10 K/min. The Nitrogen atmosphere was set 50 mL/min and the whole program lasted for 40 minutes. The samples were compared to an empty reference crucible, which was hermetically sealed and pierced, too. The calorimeter was calibrated using indium (Tm = 156.60°C).

The studies included two stages of studies. More details of the different evaluations applied are presented below (Table 3):

**Table 3:** Summary of the experiments applied during pre-formulation studies of genistein capsules.

1^st^ Study	All excipients without excipient 1
	All excipients without excipient 2
	All excipients without excipient 3
2^nd^ Study	Genistein: Excipient 1 (1:1)
	Genistein: Excipient 2 (1:1)
	Genistein: Excipient 3 (1:1)

### Analytical validation

The content of capsules in genistein was calculated and validated using Liquid Chromatography. For HPLC analysis, the capsule powder was diluted in Methanol with target concentration 10.5 ppm. Before the insertion of the solution into the vials for the analysis, the solution is filtered through 0.45 um filters (LLG, PTFE, Ø13 mm, 0,45 mm, Metrolab S.A., Athens, Greece).

$$Ps=\frac{As}{Astd}\frac{Mstd}{Ms}x\;Pstd\;x\;100\%$$ (1)

As: Peak response from the sample. Astd: Peak Response from standard solution. Mstd: Weighed mass of genistein standard. Ms: Weighed mass of genistein substance.

The purity of the capsules in Genistein was quantified based on Eq (1).

$$\frac{mg}{unit\;}=\frac{As}{Astd\;}\frac{CstdxPstd}{WxFdil}x\;MW$$ (2)

As: Peak response from the sample. Astd: Peak Response from standard solution. Cstd: Standard Solution concentration. Pstd: the purity of genistein standard.

Mw: Molecular Weight. W; the average capsule weight. Fdil: dilution factor of the standard solution.

Eq (2) is used to estimate the amount of genistein.

HPLC analysis was performed using an Agilent 1260 infinity series equipped with a multiple wavelength detector (Agilent Technologies Inc., Richardson, TX, USA). Chromatograms were integrated and analyzed using OpenLAB Chemstation (version M8301AA, Revision C.01.07 Agilent Technologies Inc., Richardson, TX, USA). The analysis of genistein was performed using a column (Zorbax Eclipse RP C18 reversed-phase, 250 mm×4.6 mm I.D., 5 μm, Agilent, Santa Clara, CA, USA) maintained at 40°C. Mobile Phase A was water, mobile Phase B was acetonitrile, and mobile Phase C was glacial acetic acid. The mobile Phases A, B, and C were mixed at a ratio of 67.5:25.0:7.5 (v/v). The flow rate was kept at 1.5 mL/min, injection volume was 10 uL and UV detection was carried out at 260 nm. The retention time of the genistein was 7.30 min.

### Stability study

After the finalization of the formulation, capsules were sent to verified outsourced laboratories at Eurofins Food Chemistry Testing (US Inc. 3301 Kinsman Blvd., Madison, WI 53704 USA). The protocol included testing in (Table 4):

**Table 4:** Summary of the stability testing of Genistein capsules prepared and formulated in R.G.C.C.’s facilities.

Conditions	Time points	Parameter
Temperature 25°C/60% Relative Humidity	Initial, 3M,6M,9M,12M,18M	Organoleptic Evaluation Disintegration (12M) Isoflavones Moisture (initial, 12M) Aerobic Plate Count USP (initial, 12M) *E*. *Coli* USP (initial) *Salmonella* USP (initial) *Staphylococcus aureus* USP (initial) Yeast and mold USP (initial, 12M)
Temperature 40°C/75% Relative Humidity	Initial, 1M, 3M, 6M,	Organoleptic Evaluation Moisture Isoflavones

### Cell culture

Two different commercial cancer cell lines were used in the present study that were purchased from ECACC (European Collection of Authenticated Cell Cultures) (Salisbury, UK). MCF7 human breast adenocarcinoma cells (luminal type) (ECACC 86012803) were cultured in RPMI 1640 supplemented with 10% FBS, 2% L-Glutamine and 2% NEAA. MDA-MB-231 human breast adenocarcinoma cells (triple negative) (ECACC 92020424) were cultured in RPMI 1640 supplemented with 15% FBS and 2% L-Glutamine. Cells were cultured in a humidified incubator at 37°C and 5% CO_2_ and passaged when cells reached 80% confluence.

### Cell viability analysis

Viability was measured with MTT for cell-metabolism activity. The viable cells were seeded at a density of 2 x 10^4^ (200 ul/well) in 96-well plate and incubated at 37°C and 5% CO_2_ for 24 h to form a cell monolayer. After 24 h of incubation, supernatant on the monolayer was discarded and 200 ul of medium and varying concentrations of the two substances were added and incubated for 24, 48 and 72 h.

After the specific time points, 20 ul of 5 mg/mL MTT Catalog#M2128 (Sigma-Aldrich, Darmstadt, Germany) in PBS Catalog#P3813 (Sigma-Aldrich, Darmstadt, Germany) was added to each well and incubated for 4 h at 37°C and 5% CO_2_. Supernatants were discarded and 100 ul of DMSO Catalog#445103 (Carlo Erbo Reagents, Barcelona, Spain) was added and the plates were incubated for 5 min 37°C and 5% CO_2_ to solubilize the formazan crystals and absorbance was measured at 560 nm and the reference wavelength was at 605 nm.

### Statistical analysis

The experiment of cell viability determination was performed in triplicates. The average absorbance was calculated for each triplicate. Subsequently, the sample measurements were corrected for the measurement of the blank. One sample t-test was used to determine differences in the mean by comparing the treated samples with the untreated controls. *P* values < 0.05 were considered to indicate a statistically significant difference. Results were calculated using the Microsoft Excel 2016.

### Bioavailability study

Targeting to launch a product improved from the market’s “golden standard”, studies were concluded with conducting pharmacokinetics study comparing R.G.C.C.’s formulation with a reference marketed formulation. The object of the study was to compare the bioavailability of 2 formulations of the dietary supplements in the male Wistar rat. This study was provided as an outsourced service by Biotrial (Biotrial Headquarters, 7–9 Rue Jean-Louis Bertrand, CS 34246, 35042 Rennes Cedex, France, Phone: +33 (0)2 99 59 91 91, Fax: +33 (0)2 99 59 91 97).

The protocol as described in the company’s final report, states that the *in vivo* study used male Wistar rats (JANVIER LABS, C.S. 4105, Saint-Berthevin F-5394, France), 293–337 g and had as housing of them cages (polysulfone cages with floor area = 1500 cm^2^) in groups of 2–4 with controlled room temperature 22±2°C, hygrometry 55±10%, light/dark cycle 12 h/12h, air replacement: 15–20 volumes/hour. Water and Food (SAFE, ref. A04) *ad libitum* except from approximately 16 hours prior to dosing until 4 hours after dosing. The test formulation (R.G.C.C. and Commercial) where both 125 mg/kg, po. The evaluation criteria were the plasma concentrations and PK parameters. At the end of the experiments, animals were sacrificed by pentobarbital overdose, given ip.

Specifically, six (6) rats received either genistein marketed formulation (125 mg/kg, n = 3) or genistein test formulation (125 mg/kg, n = 3). For both formulations, genistein capsules were opened and the required amount of powder was weighed in order to prepare one preparation per rat. As a vehicle for oral administration, 0.5% carboxymethylcellulose was used and the final formulation was at 12.5 mg/mL. For this study, animals were fasted for approximately 16 hours before dosing with the aim to avoid any baseline genistein. The volume of administration was 10 mL/kg and at the end of the study any surplus Test Item was destroyed. The dose of genistein formulations is expressed pure genistein. For the genistein marketed formulation the correction factor was determined by weighing the powder extract from the capsule, and this value was divided by the weight of genistein in each capsule, leading to a correction factor of 1.67. For the genistein test formulation, as one capsule contains 250 mg of powder including 125 mg of genistein, a correction factor of 2.0 was applied Sampling was conducting through collecting 0.5, 1, 2, 4, 8 and 24 hours after dosing blood samples of 0.5 mL and then plasma was obtained. The amount of genistein in the samples was quantified by Liquid Chromatography tandem mass spectrometry (LC-MS/MS).

The laboratory used Phoenix^®^ WinNonLin^®^ Version 7.0 for both formulations to determine from the plasma concentrations the following parameters for free genistein by standard non-compartmental methods:

- **C**_**max**_: Maximum observed plasma concentration
- **C**_**last**_: last quantifiable observed concentration
- **t**_**max**_: time to reach Cmax
- **t**_**last**_: time of occurrence of Clast
- **AUC**_**0-last**_: AUC from time zero to the last measurable plasma concentration
- **AUC**_**0-inf**_: AUC from time zero to infinity
- **t**_**1/2**_: apparent terminal elimination half-life
- **Cl**: clearance
- **Vd**: volume of distribution
- The **bioavailability** of the 2 formulations was calculated by the relative bioavailability F_rel_ calculated from the AUCs and the C_max_.

The study was conducted under EU and French animal welfare regulations for animal use in experimentation (European Directive 2010/63/EU and French decree and orders of February 1st, 2013). This experimental project is approved by the Biotrial Ethics Committee “Comité de Réflexion Ethique en Expérimentation Animale (CR2EA) (registered by the “Ministère de l’Enseignement Supérieur et de la Recherche” under No. 67)”.

The different areas under the concentration-time curve (AUC) were calculated using the linear trapezoidal summation:

- AUC_last_: Area under the plasma concentration-time curve (AUC) from time zero to the last measurable plasma concentration.
- AUC_inf_: AUC from time zero to infinity calculated as follows: AUC_inf_ = AUC_last_ + (C_last/_λ_z_), where λ_z_: apparent terminal rate constant calculated by log-linear regression of the terminal segment of the drug plasma concentration versus time curve
- t_1/2_: apparent terminal elimination half-life calculated as follow: t_1/2_ = *ln*(2)/λ_z_
- Cl: clearance calculated as follows: Cl = Dose/AUC_inf_
- Vd: volume of distribution calculated as follows: Vd = Cl/λ_z_

As no blood sampling before dosing was performed, T0 values were fixed at 0 μM. In addition, BLQ levels were considered to LLOQ/2, i.e, 0.5 ng/mL, in order to enable the calculation of PK parameters.

Descriptive statistics were performed for all parameters. Time profile plots were prepared on linear and log/linear coordinates for each animal, as well as arithmetic mean (±SD) for both formulations. No statistical analysis was performed.

Finally, the bioavailability of the 2 formulations was evaluated by calculation of the relative bioavailability F_rel_ calculated from the AUCs and the C_max_ as follows:

$${\text{F}}_{\text{rel}}={\text{AUC}}_{\text{test}}/{\text{AUC}}_{\text{ref}}{\;\text{for}\;\text{AUC}}_{\text{last}}{\text{and}\;\text{AUC}}_{\text{inf}}({\text{F}}_{\text{rel1}}{\text{and}\;\text{F}}_{\text{rel2}},\;\text{respectively})\;{\text{and}\;\text{F}}_{\text{rel3}}={\text{C}}_{\text{max}(\text{test})}/{\text{C}}_{\text{max}(\text{ref})}$$

## Results

### Formulation of genistein capsules

After conducting a series of experiments, the best formulation of Genistein capsules was determined upon the results described in the following sections, after a series of trials and experiments.

### Flowability

One of the most vital attributes of the powder is the way it flows and dispenses. It is a factor with an impact not only during the formulation development in the laboratory but also in the manufacturing stages. For each measurement, around 6.5 g of mixture were used. The results of the flow properties of the powders developed are presented below (Table 5).

**Table 5:** Results of the flow properties of the different formulations developed.

Formulation Code	Height	Radius	Angle of repose	Result
1	3	3.625	39.5°	Fair (aid not needed)
2	3	4	36.9°	Fair (aid not needed)
3	3	3.3	42.3°	Passable (may hang up)
4	3	3.35	41.8°	Passable (may hang up)
5	3	3.875	37.7°	Fair (aid not needed)

### Dissolution-disintegration

One test that can offer directly at hand critical safety data on drug behavior and bioavailability, is disintegration. Oral dosage units need to be in a solution in order to be absorbed and act effectively. After the administration of an oral pharmaceutical dosage unit, the first step towards solution is disintegration. In this stage, pharmaceutical dosage units break into granules or particles. Time and rate during this process might differ and be depended upon the scope and type of formulation.

The disintegration tests were all in general satisfying and capsules seems to behave properly in these conditions. The results are summarized at Table 6, where it is apparent that formulation 2, had the best disintegration behavior.

**Table 6:** Results after the disintegration test of the capsules in Phosphate Buffer Solution (PBS), pH 6.8.

Formulation Code	Disintegration time (min.)
1	10
**2**	**8**
3	9.3
4	10.3
5	9

The parameter that controls the oral bioavailability *in vitro* of oral pharmaceutical dosage units, is dissolution. The rate that the active pharmaceutical ingredient can dissolute in the aqueous media is a crucial factor since it is connected to its instinct absorption [29]. In this case, the five different pharmaceutical forms were tested in 3% SDS aq. Solution. In each vessel, the basket contained one capsule and the temperature was stable at 37°C. For each formulation form, there is an extra vessel containing the excipients only in the same quantities in order to use as reference. Graph 1 and Table 7 show the dissolution percentage per formulation unit. Each experiment was repeated at least in triplicate and the results at Table 3 are the average of them.

**Table 7:** Quantity (%) of the dissoluted API (Genistein) during different sampling intervals.

Time (minutes)	30	45	60	75	90
Formulation					
**1**	26,8	59,7	76,0	82,4	83,4
**2**	53,3	85,7	95,8	100,0	100,0
**3**	44,4	68,0	73,3	78,2	80,9
**4**	48,6	70,2	77,9	81,3	84,2
**5**	43,9	66,3	74,6	78,3	80,5

The solution used for this experiment is 3% SDS aqueous solution.

As it is seen after the quality controls studies that were conducted, only formulation 2 completed the tests with success.

*Thus, the next studies, were conducted only on this formulation*.

### Differential scanning calorimetry

Multiple studies were conducted between the API and the excipients. The excipients were tested either in mixtures between each other either with the API. One of the best methods to check incompatibilities and possible by-products or even degradants is to examine the thermal properties.

The thermograms obtained and presented at Graphs 2 and 3, show that no incompatibilities or unexpected events were caused with these combinations and the finalization of the ingredients was verified. Genistein did not present any by-product and its characteristic endothermic peak above 300°C, is apparent but transferred at lower temperature in the mixtures.

#### Quantification of Genistein by HPLC

At Graph 4, the chromatograph of R.G.C.C.’s Genistein capsule is presented. It is obvious that pure Genistein is present in the product and the quantification of it, showed 100% API in the capsule.

#### Stability testing

The results of the stability testing conducted at Eurofins Food Chemistry Testing are summarized at the tables below (Tables 8 and 9, accordingly). Both the real time and the accelerated stability testings agreed that the amount of genistein inside the capsules as well as the organoleptic characteristics of the product describe a stable product with an expire life of 2 years. Each measurement used two capsules of 30 mg Genistein each.

**Table 8:** Brief summary of the results of the accelerated stability testing (40°C/ 60% RH).

	Initial	1 Month	3 Months	6 Months
**Isoflavones by HPLC (mg/mL)**	63.1	66.5	66.0	61.4
**Moisture (%)**	4.49	4.20	4.85	4.43
**Organoleptic evaluation**	Clear capsule with off-white powder fill White plastic bottle with white lid Mild unidentifiable odor	Clear capsule with off-white powder fill White plastic bottle with white lid Mild unidentifiable odor	Clear capsule with off-white powder fill White plastic bottle with white lid Mild unidentifiable odor	Clear capsule with off-white powder fill White plastic bottle with white lid Mild unidentifiable odor

**Table 9:** Brief summary of the results of real time stability testing (25°C/ 75% RH).

	Initial	3 Months	6 Months	9 Months	12 Months	18 Months
**Isoflavones by HPLC (mg/mL)**	63.1	66,7	66,4	61.3	63.0	67.3
**Moisture (%)**	4.49	-	-	-	4.57	-
**Organoleptic evaluation**	Clear capsule with off-white powder fill White plastic bottle with white lid Mild unidentifiable odor	Clear capsule with off-white powder fill White plastic bottle with white lid Mild unidentifiable odor	Clear capsule with off-white powder fill White plastic bottle with white lid Mild unidentifiable odor	Clear capsule with off-white powder fill White plastic bottle with white lid Mild unidentifiable odor	Clear capsule with off-white powder fill White plastic bottle with white lid Mild unidentifiable odor	Clear capsule with off-white powder fill White plastic bottle with white lid Mild unidentifiable odor
**Aerobic Plate Count (CFU/g)**	10	-	-	-	<10	-
**Salmonella USP (CFU/g)**	Absent/10g	-	-	-	-	-
**E.Coli USP (CFU/g)**	Absent/10g	-	-	-	-	-
**Staphylococcus Aureus (CFU/g)**	Absent/10g	-	-	-	-	-
**Yeasts and Mold (CFU/g)**	<10	-	-	-	<10	-
**Disintegration (minutes)**	<30	-	-	-	<30	-

### Effect of Genistein R.G.C.C. and Genistein Commercial on the proliferation of the human cancer cell lines

The *in vitro* activity of Genistein R.G.C.C. compared to R.G.C.C. Commercial was determined with MTT assay. The effect of those two substances on the two human breast cancer cell lines was assessed with the concentration range 0.001, 0.01, 0.1, 1, 10 and 100 uM. The cells were incubated with each substance for 24, 48 and 72 h. Untreated control was also included in the experiments.

**Fig 1:**
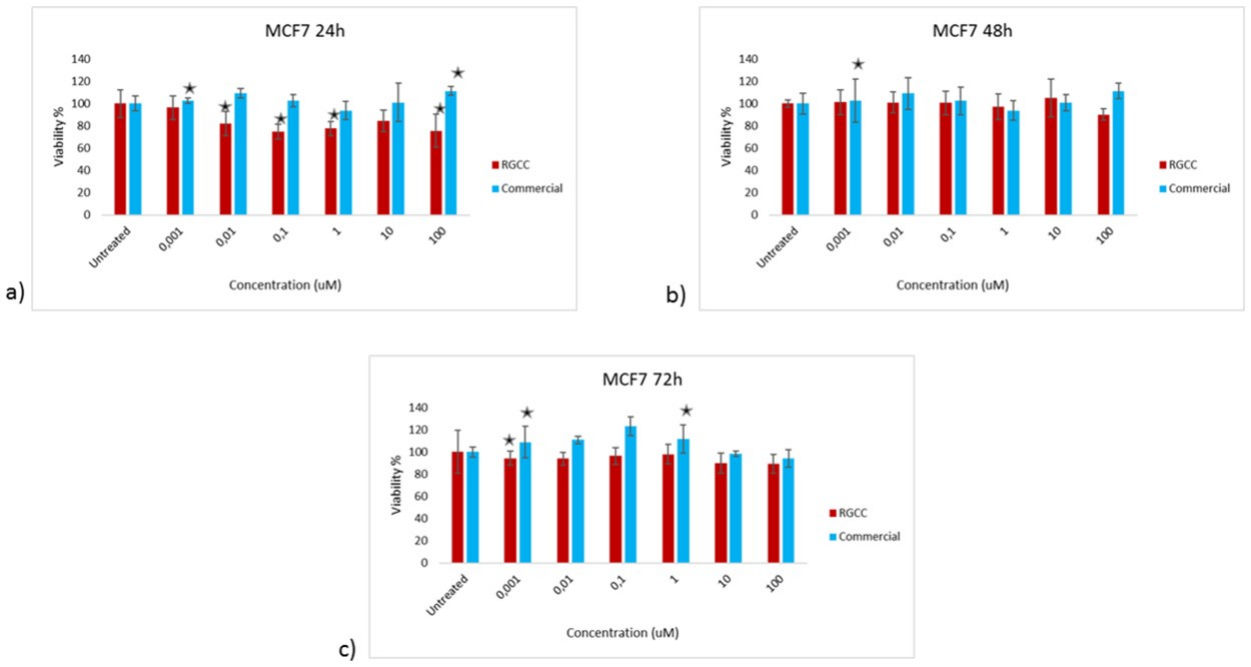
Viability of MCF7 cells after the treatment with various concentrations of genistein R.G.C.C. and genistein commercial. MTT results at a) 24 h, b) 48 h and c) 72 h. The * represents the statistically significant results *P*-value <0.05.

Hormone sensitive human breast adenocarcinoma cell line MCF7 was treated with the two substances and the proliferation assay was performed at the three time points. Genistein R.G.C.C. had a statistically significant effect on the proliferation of MCF7 cells at 24 h and the viability of the cells was decreased by 25% even at low concentrations of the substance (0.1 and 1 uM) (Fig 1). Genistein Commercial did not show similar effect on the same cell line. There was no statistically significant decrease on the viability of MCF7 cells after 24 h of the treatment with Genistein Commercial. Moreover, there was no significant effect at 48 h and 72 h of both substances against MCF7 cells.

**Fig 2:**
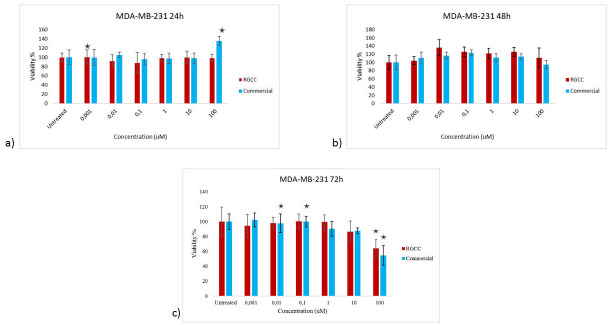
Viability of MDA-MB-231 cells after the treatment with various concentrations of genistein R.G.C.C. and genistein commercial. MTT results at a) 24 h, b) 48 h and c) 72 h. The * represents the statistically significant results *P*-value <0.05.

On the other hand, both substances appeared to have no statistically significant effect on the proliferation of the triple negative human breast adenocarcinoma cell line MDA-MB-231 (Fig 2). There was only a significant decrease of cell viability at 72h with the highest concentration, 100uM, of the two substances.

### Bioavailability

**Fig 3:**
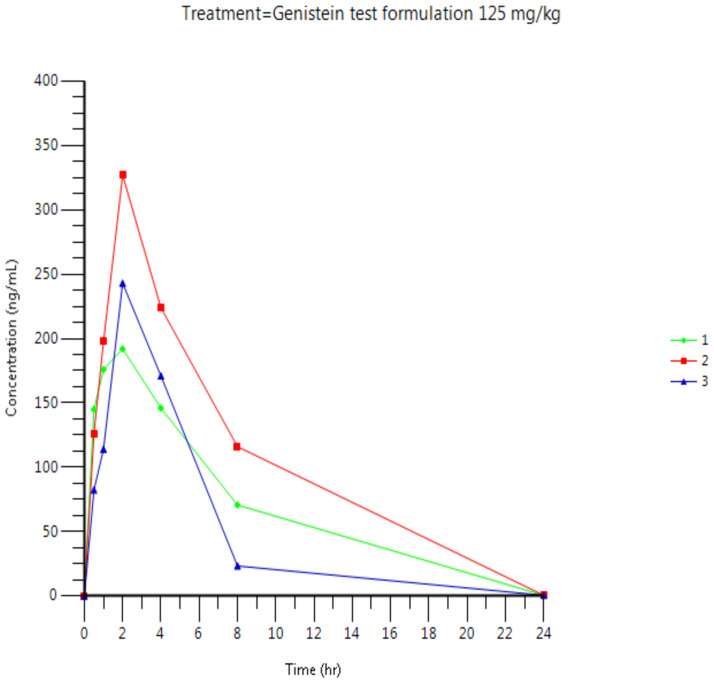
Individual plasma concentration of genistein after oral administration of genistein (125 mg/kg) as test formulation in male Wistar rats.

**Fig 4:**
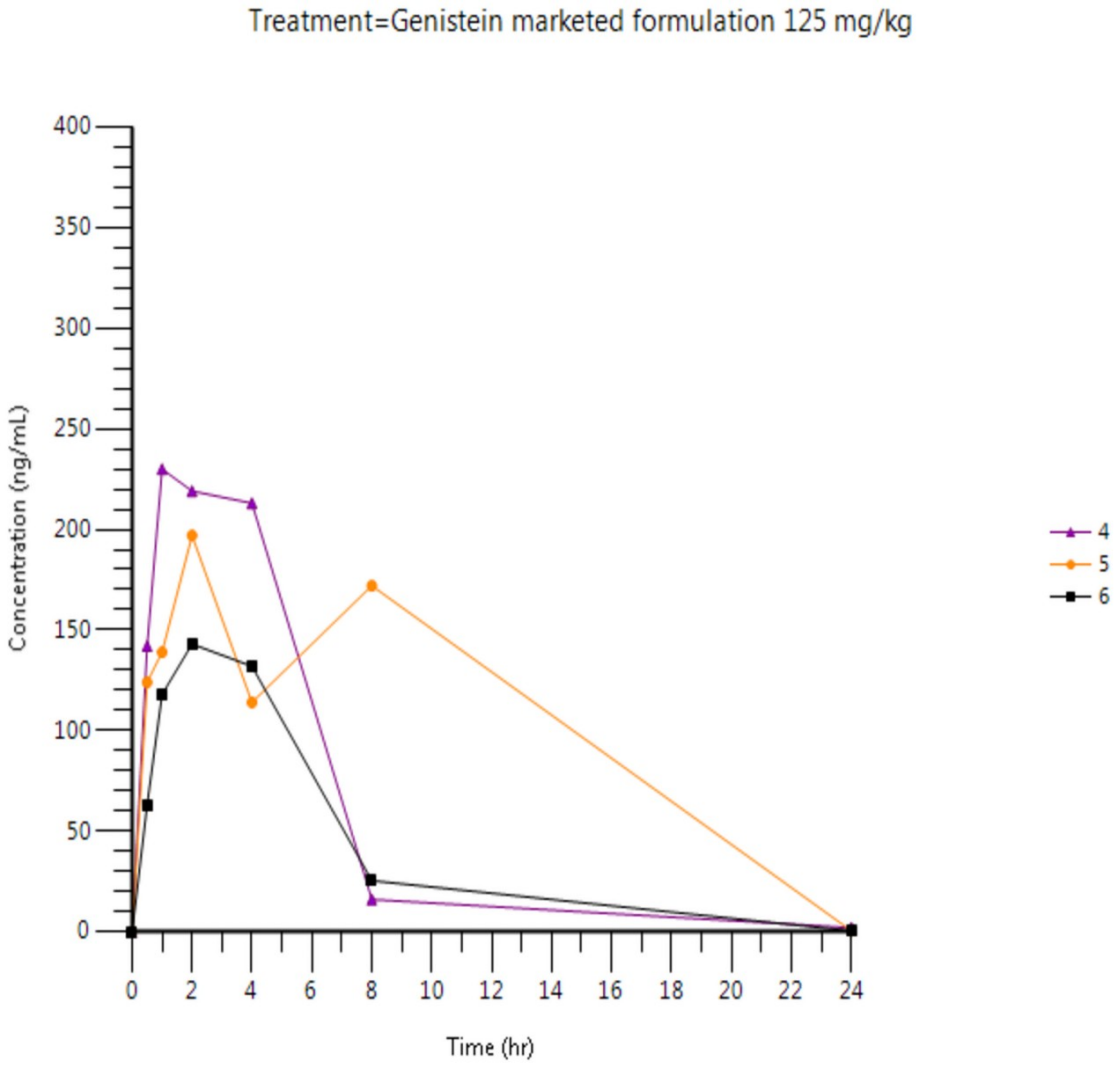
Individual plasma concentration of genistein after oral administration of genistein (125 mg/kg) as marketed formulation in male Wistar rats.

**Fig 5:**
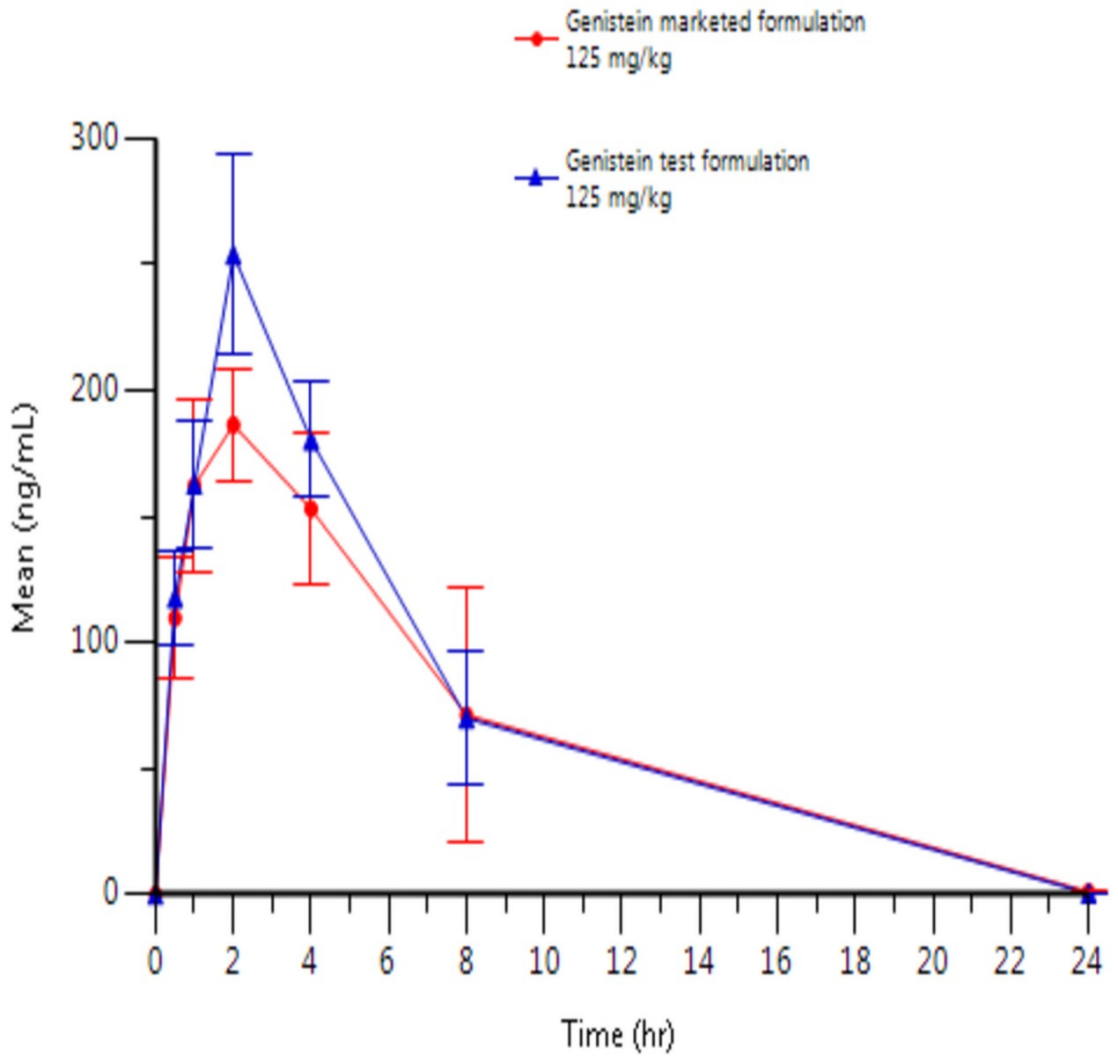
Arithmetic mean (±SD) plasma concentration of genistein after oral administration of genistein (125 mg/kg) as marketed formulation or test formulation in male Wistar rats.

Plasma concentrations are presented in Fig 3 (individual results for the test formulation), Fig 4 (individual results for the reference formulation) and Fig 5 (mean results for both formulations). Tables 10 and 11 present Arithmetic mean (CV%) plasma concentration (ng/mL) of Genistein and relative bioavailability, respectively.

**Table 10:** Arithmetic mean (CV%) plasma concentration (ng/mL) of genistein after oral administration of the genistein test and marketed formulation (125 mg/kg) in male Wistar rats.

	Genistein 125 mg/kg po test formulation—Arithmetic mean results		Genistein 125 mg/kg po marketed formulation—Arithmetic mean results	
Time (h)	Mean	CV%	Mean	CV%
0*	0	.	0	.
0.5	117.8	27.3	109.7	37.7
1	162.7	26.8	162.3	36.7
2	254.0	26.8	186.3	21.0
4	180.3	22.1	153.0	34.5
8	70.0	66.2	71.2	122.9
24	0.5	0	1.007	87.2
**Plasma parameters**				
**C**_**max**_ **(ng/mL)**	254	26.8	190	23.1
**t**_**max**_ **(h)**	2.02 ^a^	[2.0–2.03] ^a^	2.00 ^a^	[1.00–2.00] ^a^
**AUC**_**last**_ **(ng.h/mL)**	1806	36.8	1635	48.9
**AUC**_**inf**_ **(ng.h/mL)**	1808	36.8	1639	48.7
**t**_**1/2**_ **(h)**	2.36	6.5	2.71	17.5
**t**_**last**_ **(h)**	23.98 ^a^	[23.98–24.0] ^a^	24.00 ^a^	[23.98–24.02] ^a^
**C**_**last**_ **(ng/mL)**	0.5	0	1.007	87.2
**Cl (mL/h/kg)**	75357	34.2	88381	43.6
**Vd (mL/kg)**	260292	39.9	351090	46.3

Actual sampling times were used to perform the PK analysis.

ª Median and [min-max].

**Table 11:** Relative Bioavailability of test genistein formulation versus marketed genistein formulation.

Parameters	Calculation	Results
**F**_**rel1**_	AUC_last(test)/_AUC_last(ref)_	1.10
**F**_**rel2**_	AUC_inf(test)/_AUC_inf(ref)_	1.10
**F**_**rel3**_	C_max(test)_/C_max(ref)_	1.34

Following single oral administration of 125 mg/kg genistein as reference (marketed formulation) or test formulation, genistein was detected at concentrations above the lower limit of quantitation (i.e., LLOQ of 1.00 ng/mL) up to 8 hours post-dose for all rats, except rat No.4 who presented a t_last_ at 24 hours.

Following oral administration of 125 mg/kg genistein as reference formulation, mean peak plasma levels (C_max_) and exposure (AUClast and AUCinf) were 190 ng/mL, 1635 ng.h/mL and 1639 ng.h/mL, respectively. A higher C_max_, AUC_las_t and AUC_inf_ were observed after oral administration of 125 mg/kg genistein as test formulation with mean C_max_, AUC_las_t and AUC_inf_ values of 254 ng/mL, 1806 ng.h/mL and 1808 ng.h/mL, respectively. A 34% higher C_max_ and 10% higher AUCs were observed for the test formulation compared to the reference formulation (i.e, with F_rel1_ and F_rel2_ of 1.10 and F_rel3_ of 1.34, respectively).

The evaluation of the elimination half-life of all rats show similar results for both reference and test formulations with mean elimination half-lives of 2.71 and 2.36 hours following administration of the marketed and test formulation, respectively.

The inter-individual variability of the main PK parameters (C_max_ and AUCs) was moderate, with CV% ranging between 23.1 and 48.9%.

## Discussion

Pharmacopoeia requires standard procedures with the aim to reassure the quality of pharmaceutical products. These guidelines may cover mostly drugs and products with direct therapeutic action, but it is up to the company’s philosophy to follow the same guidelines and procedures for non-drug pharmaceutical products, e.g. dietary supplements. In this case, it was chosen to follow the same steps to determine the quality of the in-house product and their properties.

Flavonoids can inhibit proliferation and induce apoptosis of cancer cells. One of the major isoflavones is genistein, along with daidzein and glycitein. It was chosen to formulate Genistein in capsules, a patient friendly pharmaceutical dosage form with enhanced pharmacokinetic profile and proven action, *in vitro* and *in vivo*. Quality control showed positive results, without any implications in formulation and an impressive pharmaceutical profile, since disintegration and dissolution times were within the limits. It is vital to state here that in the case of Biopharmaceutical (BCS) Class II compounds (compounds with low solubility but high permeability), this is rather rare. Stability protocols, both real time and accelerated time conditions, showed satisfactory results throughout the study and at this point, it is worth mentioning that the protocol is still on going.

Isoflavones possess anticarcinogenic and antioxidant properties that lead to their chemopreventive activity against various cancer types [32]. According to the *in vitro* activity determination of Genistein R.G.C.C., it was clear that the substance significantly decreases the proliferation of the hormone sensitive MCF7 cell line at 24 h of treatment with the specific substance. The formulation of Genistein R.G.C.C. has given enhanced properties to the substance as comparing to Genistein Commercial, which did not show any effect on the viability of MCF7 cells, Genistein R.G.C.C. appeared to be effective even at very low concentrations. Genistein is a phytoestrogen that interacts with the estrogen receptors [33]. Triple negative human breast adenocarcinoma cell line MDA-MB-231 was lacking of the specific receptors and it could be easily confirmed with the viability analysis that no effect of both Genistein substances was observed on the specific cells.

Flavonoids can inhibit proliferation and induce apoptosis of cancer cells. One of the major isoflavones is genistein, along with daidzein and glycitein. Isoflavones possess anticarcinogenic and antioxidant properties that lead to their chemopreventive activity against various cancer types [32]. According to the *in vitro* activity determination of Genistein R.G.C.C., it was clear that the substance significantly decreases the proliferation of the hormone sensitive MCF7 cell line at 24 h of treatment with the specific substance. The formulation of Genistein R.G.C.C. has given enhanced properties to the substance as comparing to Genistein Commercial, which did not show any effect on the viability of MCF7 cells, Genistein R.G.C.C. appeared to be effective even at very low concentrations. Genistein is a phytoestrogen that interacts with the estrogen receptors [33]. Triple negative human breast adenocarcinoma cell line MDA-MB-231 was lacking of the specific receptors and it could be easily confirmed with the viability analysis that no effect of both Genistein substances was observed on the specific cells.

Analogous results were obtained from the pharmacokinetics study. The improved R.G.C.C. formulation of Genistein compared to the market’s most commercial product presented imposing results. The C_max_ of R.G.C.C.’s product was 34% higher than the commercial one with 10% higher AUC. This is significantly higher maximum observed plasma concertation and a slight but important increase in exposure.

## Strengths and limitations

This study provides a comprehensive and complete background, in the field of pharmaceutical technology and product development. Since we already experience the era of dietary supplements and consumption of natural products in our daily diet, it is high time for companies to concern themselves with analytical development of products. Dietary supplements have a strong role in our lifestyle, thus their quality and efficacy must be thoughtfully evaluated. These experiments offer a broad scientific view on the quality aspects of the product, not only chemically but also biologically. However, as long as this study does not include data from human beings, either healthy or patients, it can have a role as a starting point of a new research field.

## Conclusion

This study proved that changes in formulation could affect the efficacy of a dosage form and enhance its properties. This resulted in an improved product with proven activity. The enhanced properties, made the product exhibited improved *in vitro* behavior and great pharmacokinetic profile.

Based on the literature information on Isoflavones and Genistein, doctors and physicians could recommend the daily intake of Genistein. An overall review on its action combined with therapeutic agents, can be a useful tool for clinical cases.

## Supporting information

S1 Fig Dissolution of 5 different formulations of genistein capsules in 3% SDS aq. solution. (TIF)

S2 Fig DSC thermograms of excipients in different combinations; (1) All excipients, (2) All excipients except from excipient 1, (3) All excipients except from excipient 2, (4) All excipients except from excipient 3. (TIF)

S3 Fig DSC thermograms of mixtures of Genistein with each of the excipients separately; (1) Pure Genistein, (2) Genistein with excipient 1 (1:1), (3) Genistein with excipient 2 (1:1), (4) Genistein with excipient 3 (1:1). (TIF)

S4 Fig High-performance liquid chromatography chromatographs of research genetic cancer centre dietary supplement. ppm). All chromatograms obtained by Zorbax Eclipse RP C18 reversed-phase column (250 mm×4.6 mm, 5 μm), mobile phase of water: acetonitrile: glacial acetic acid (67.5:25.0:7.5) and flow rate of 1.5 mL/min.
(TIF)

S1 File (RAR)

## References

[1] de Oliveira Stela R., Taveira Stephânia F., Marreto Ricardo N., Valadares Marize C., Diniz Danielle G.A., Lima Eliana M., Preparation and characterization of solid oral dosage forms containing soy isoflavones, Revista Brasileira de Farmacognosia, Volume 23, Issue 1, 2013, 175–181.

[2] Seung-Hyun LEE, Young Heui KIM, Heui-Jong YU, Nam-Suk CHO, Tae-Hyun KIM, Dong-Chool KIM, et al (2007) Enhanced Bioavailability of Soy Isoflavones by Complexation with β-Cyclodextrin in Rats, Bioscience, Biotechnology, and Biochemistry, 71:12, 2927–2933. 10.1271/bbb.70296 18071265

[3] Setchell KD, Brzezinski A, Brown NM, Desai PB, Melhem M, Meredith T, et al. Pharmacokinetics of a slow-release formulation of soybean isoflavones in healthy postmenopausal women. J Agric Food Chem. 2005 3 23;53(6):1938–44. 10.1021/jf0488099 15769117

[4] Sarkar FH, Adsule S, Padhye S, Kulkarni S, Li Y. The role of genistein and synthetic derivatives of isoflavone in cancer prevention and therapy. Mini Rev Med Chem. 2006 4;6(4):401–7. 10.2174/138955706776361439 16613577

[5] Pavese JM, Farmer RL, Bergan RC. Inhibition of cancer cell invasion and metastasis by genistein. Cancer Metastasis Rev. 2010 9;29(3):465–82. 10.1007/s10555-010-9238-z 20730632 PMC2933845

[6] Vaishampayan U, Hussain M, Banerjee M, Seren S, Sarkar FH, Fontana J, et al. Lycopene and soy isoflavones in the treatment of prostate cancer. Nutr Cancer. 2007;59(1):1. 10.1080/01635580701413934 17927495

[7] Kim SH, Kim CW, Jeon SY, Go RE, Hwang KA, Choi KC. Chemopreventive and chemotherapeutic effects of genistein, a soy isoflavone, upon cancer development and progression in preclinical animal models. Lab Anim Res. 2014 12;30(4):143–50. 10.5625/lar.2014.30.4.143 25628724 PMC4306701

[8] Vlachou Ioanna & Papasotiriou Ioannis. (2020). Quality Assessment And Quantification Of Genistein In Dietary Supplements By High-Performance Liquid Chromatography, Quantitative Nuclear Magnetic Resonance, And Two-Dimensional Diffusion Ordered Spectroscopy 1h. Asian Journal of Pharmaceutical and Clinical Research. 132–142.

[9] Spagnuolo C, Russo GL, Orhan IE, Habtemariam S, Daglia M, Sureda A, et al. Genistein and cancer: current status, challenges, and future directions. Adv Nutr. 2015 7 15;6(4):408–19. 10.3945/an.114.008052 26178025 PMC4496735

[10] Steensma A, Faassen-Peters MA, Noteborn HP, Rietjens IM. Bioavailability of genistein and its glycoside genistin as measured in the portal vein of freely moving unanesthetized rats. J Agric Food Chem. 2006 10 18;54(21):8006–12. 10.1021/jf060783t 17032002

[11] Lee JY, Kim HS, Song YS. Genistein as a Potential Anticancer Agent against Ovarian Cancer. J Tradit Complement Med. 2012 4;2(2):96–104. 10.1016/s2225-4110(16)30082-7 24716121 PMC3942921

[12] Allred CD, Allred KF, Ju YH, Virant SM, Helferich WG. Soy diets containing varying amounts of genistein stimulate growth of estrogen-dependent (MCF-7) tumors in a dose-dependent manner. Cancer Res. 2001 7 1;61(13):5045–50. 11431339

[13] Peterson Greg, Barnes Stephen, Genistein inhibition of the growth of human breast cancer cells: Independence from estrogen receptors and the multi-drug resistance gene, Biochemical and Biophysical Research Communications, Volume 179, Issue 1, 1991, 661–667. 10.1016/0006-291x(91)91423-a 1883387

[14] Lamartiniere CA. Protection against breast cancer with genistein: a component of soy. Am J Clin Nutr. 2000 6;71(6 Suppl):1705S–7S; discussion 1708S-9S. 10.1093/ajcn/71.6.1705S 10837323

[15] Ziaei S, Halaby R. Dietary Isoflavones and Breast Cancer Risk. Medicines (Basel). 2017 4 7;4(2):18. 10.3390/medicines4020018 28930233 PMC5590054

[16] Perabo FG, Von Löw EC, Ellinger J, von Rücker A, Müller SC, Bastian PJ. Soy isoflavone genistein in prevention and treatment of prostate cancer. Prostate Cancer Prostatic Dis. 2008;11(1):6–12. 10.1038/sj.pcan.4501000 17923857

[17] Yang Z., Kulkarni K., Zhu W., & Hu M. (2012). Bioavailability and pharmacokinetics of genistein: mechanistic studies on its ADME. Anti-cancer agents in medicinal chemistry, 12(10), 1264–1280. 10.2174/187152012803833107 22583407 PMC4010305

[18] Davis J. N., Kucuk O., & Sarkar F. H. (1999). Genistein inhibits NF-kappa B activation in prostate cancer cells. Nutrition and cancer, 35(2), 167–174. 10.1207/S15327914NC352_11 10693171

[19] Peterson G. and Barnes S. (1993), Genistein and biochanin A inhibit the growth of human prostate cancer cells but not epidermal growth factor receptor tyrosine autophosphorylation. Prostate, 22: 335–345. 10.1002/pros.2990220408 8497428

[20] Lakshman M, Xu L, Ananthanarayanan V, Cooper J, Takimoto CH, Helenowski I, et al. Dietary genistein inhibits metastasis of human prostate cancer in mice. Cancer Res. 2008 3 15;68(6):2024–32. 10.1158/0008-5472.CAN-07-1246 18339885

[21] Perabo FG, Von Löw EC, Ellinger J, von Rücker A, Müller SC, Bastian PJ. Soy isoflavone genistein in prevention and treatment of prostate cancer. Prostate Cancer Prostatic Dis. 2008;11(1):6–12. 10.1038/sj.pcan.4501000 17923857

[22] Tang J, Xu N, Ji H, Liu H, Wang Z, Wu L. Eudragit nanoparticles containing genistein: formulation, development, and bioavailability assessment. Int J Nanomedicine. 2011; 6:2429–35. 10.2147/IJN.S24185 22072878 PMC3205137

[23] Barnes S. “Effect of genistein on in vitro and in vivo models of cancer.” The Journal of nutrition vol. 125,3 Suppl (1995): 777S–783S. 7884564 10.1093/jn/125.3_Suppl.777S

[24] Hussain M, Banerjee M, Sarkar FH, Djuric Z, Pollak MN, Doerge D, et al. Soy isoflavones in the treatment of prostate cancer. Nutr Cancer. 2003;47(2):111–7. 10.1207/s15327914nc4702_1 15087261

[25] Russo M, Russo GL, Daglia M, Kasi PD, Ravi S, Nabavi SF, et al. Understanding genistein in cancer: The "good" and the "bad" effects: A review. Food Chem. 2016 4 1;196:589–600. 10.1016/j.foodchem.2015.09.085 26593532

[26] Hun Lee J., Shu L., Fuentes F., Su Z. Y., & Tony Kong A. N. (2013). Cancer chemoprevention by traditional chinese herbal medicine and dietary phytochemicals: targeting nrf2-mediated oxidative stress/anti-inflammatory responses, epigenetics, and cancer stem cells. Journal of traditional and complementary medicine, 3(1), 69–79. 10.4103/2225-4110.107700 24716158 PMC3924975

[27] Taylor CK, Levy RM, Elliott JC, Burnett BP. The effect of genistein aglycone on cancer and cancer risk: a review of in vitro, preclinical, and clinical studies. Nutr Rev. 2009 7;67(7):398–415 10.1111/j.1753-4887.2009.00213.x 19566600

[28] Cohen R, Schwartz B, Peri I, Shimoni E. Improving bioavailability and stability of genistein by complexation with high-amylose corn starch. J Agric Food Chem. 2011 7 27;59(14):7932–8. 10.1021/jf2013277 21688810

[29] Motlekar N., Khan M.A. and Youan B.-B.C. (2006), Preparation and characterization of genistein containing poly(ethylene glycol) microparticles. J. Appl. Polym. Sci., 101: 2070–2078.

[30] Kesarwani K., Gupta R., & Mukerjee A. (2013). Bioavailability enhancers of herbal origin: an overview. Asian Pacific journal of tropical biomedicine, 3(4), 253–266. 10.1016/S2221-1691(13)60060-X 23620848 PMC3634921

[31] Suk Hyung Kwon Myung Joo Kang, Jin Seo Huh Kyung Wook Ha, Jeong Rai Lee Sang Kil Lee, et al. Comparison of oral bioavailability of genistein and genistin in rats, International Journal of Pharmaceutics, Volume 337, Issues 1–2, 2007, Pages 148–154, ISSN 0378-5173. 10.1016/j.ijpharm.2006.12.046 17280808

[32] Sanaei M, Kavoosi F, Valiani A, Ghobadifar MA. Effect of genistein on apoptosis and proliferation of hepatocellular Carcinoma Hepa1-6 Cell Line. Int J Prev Med 2018;9:12. 10.4103/ijpvm.IJPVM_249_16 29541427 PMC5843956

[33] Pons D.G., Nadal-Serrano M., Torrens-Mas M., Oliver J. and Roca P. (2016), The Phytoestrogen Genistein Affects Breast Cancer Cells Treatment Depending on the ERα/ERβ Ratio. J. Cell. Biochem., 117: 218–229. 10.1002/jcb.25268 26100284

