# Supplementary figures and images for: Genistein as a dietary supplement; formulation, analysis and pharmacokinetics study
Source: PLoS One. 2021 Apr 27;16(4):e0250599. doi: 10.1371/journal.pone.0250599 (PMC8078810; doi:10.1371/journal.pone.0250599)

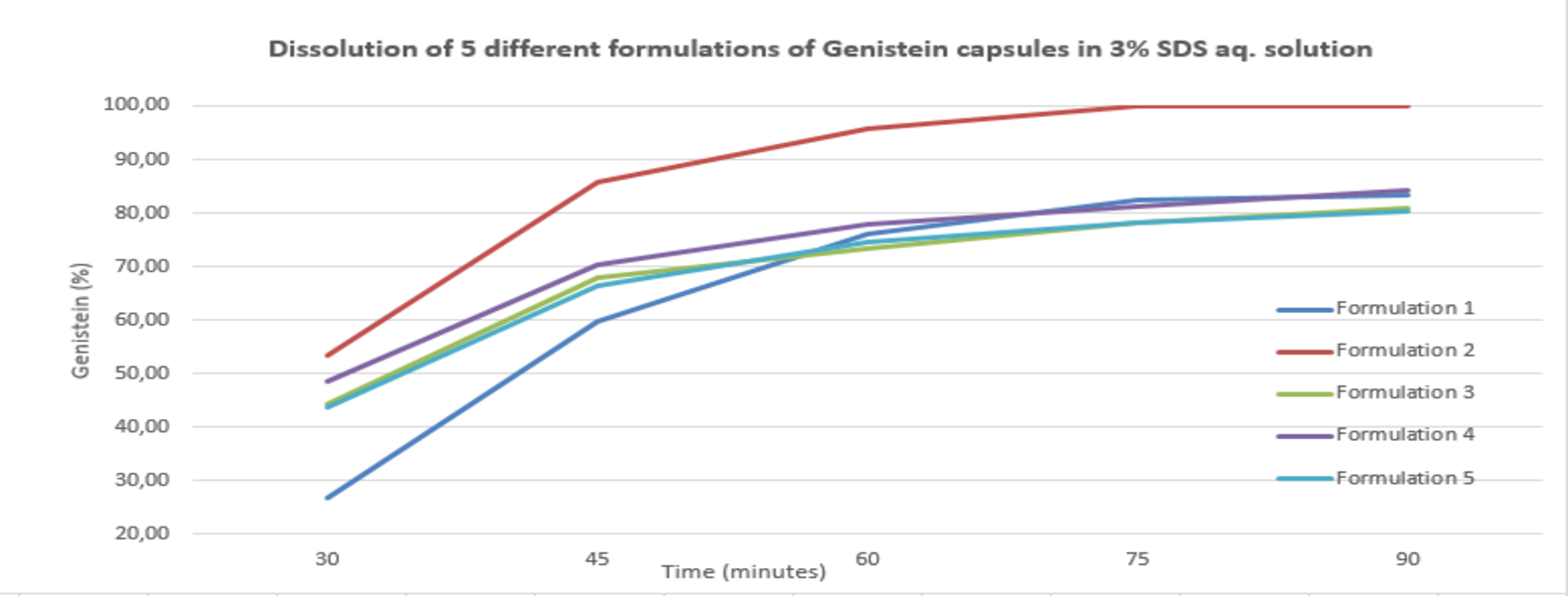

Supplement: S1 Fig — (TIF) [file pone.0250599.s001.tif]

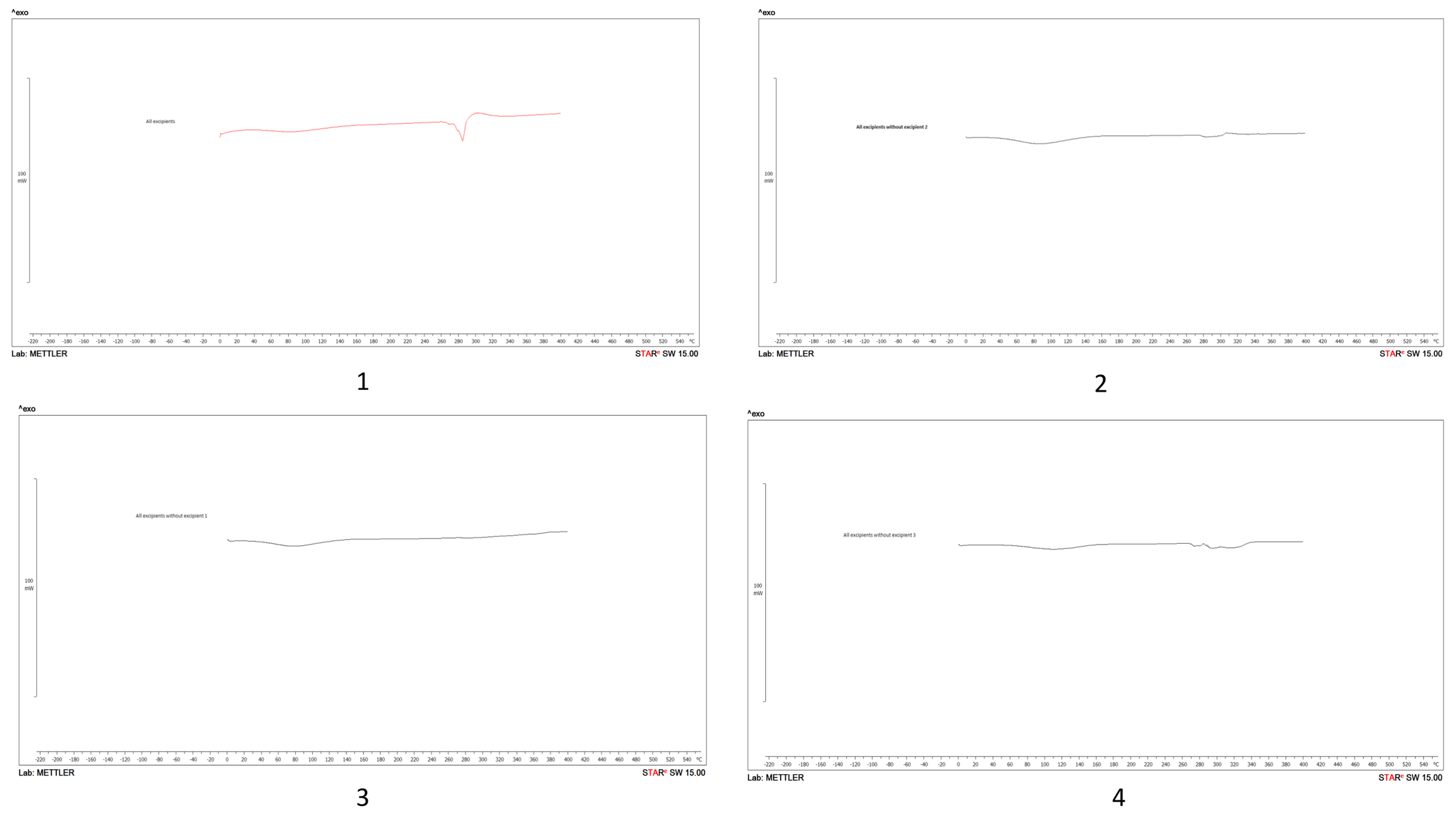

Supplement: S2 Fig — (TIF) [file pone.0250599.s002.tif]

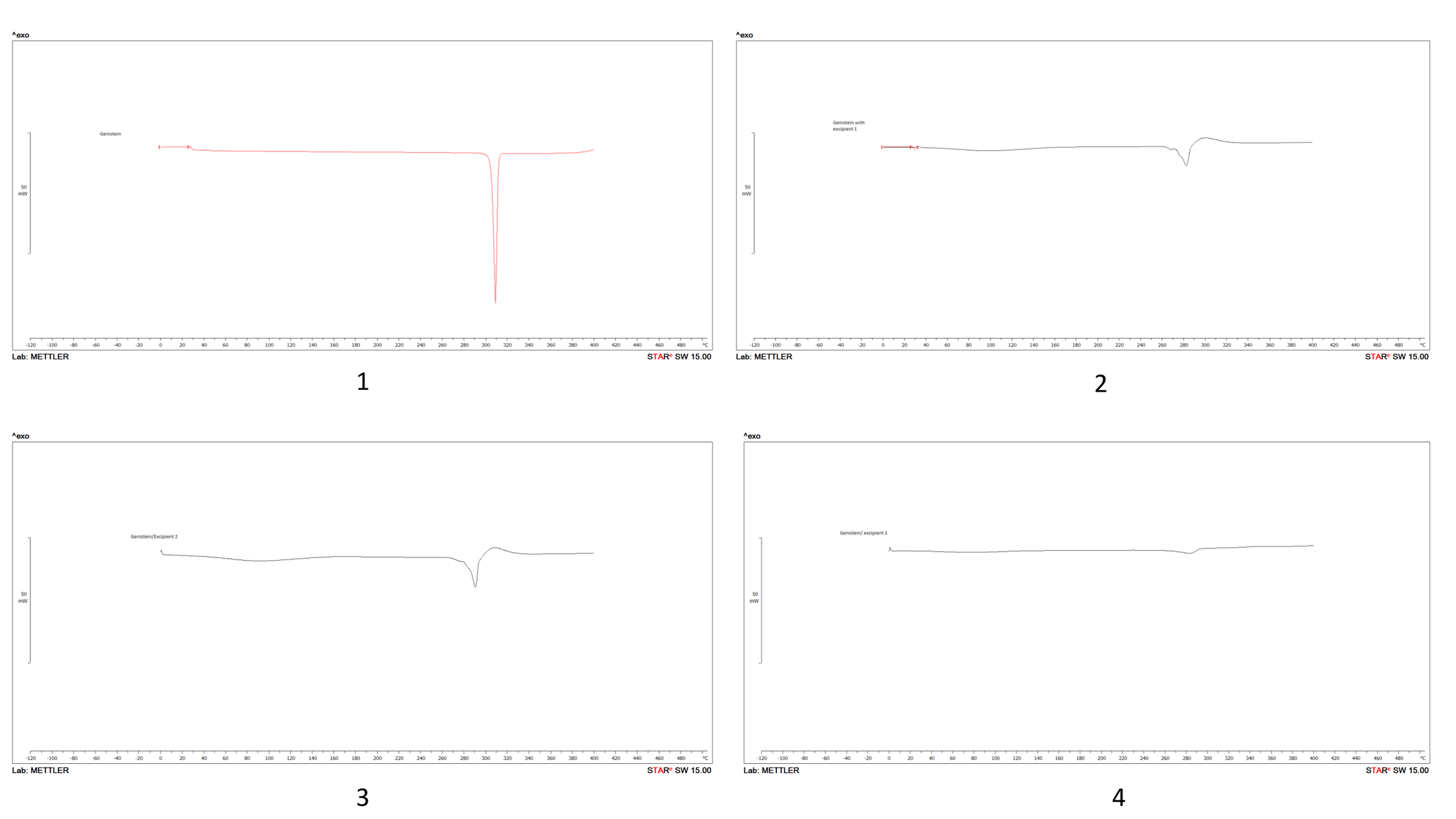

Supplement: S3 Fig — (TIF) [file pone.0250599.s003.tif]

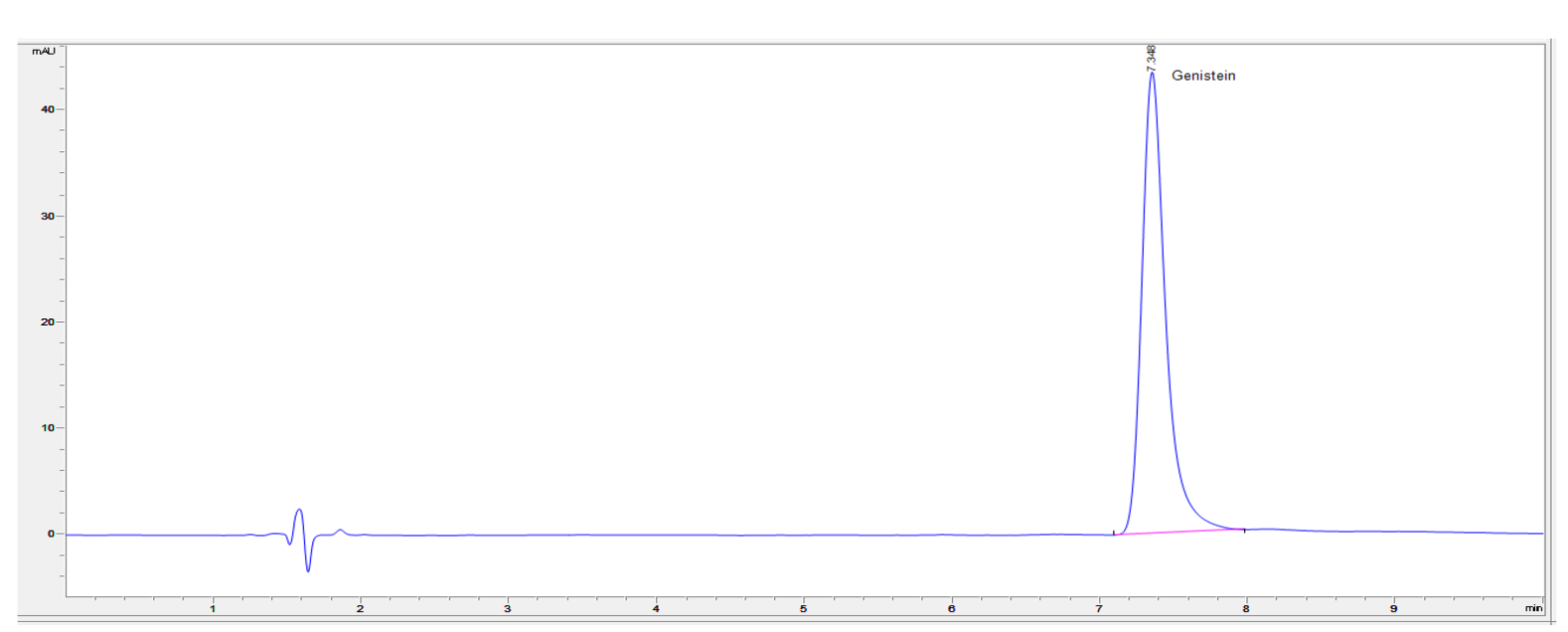

Supplement: S4 Fig — All chromatograms obtained by Zorbax Eclipse RP C18 reversed-phase column (250 mm×4.6 mm, 5 μm), mobile phase of water: acetonitrile: glacial acetic acid (67.5:25.0:7.5) and flow rate of 1.5 mL/min. (TIF) [file pone.0250599.s004.tif]
